# Inferring potential non-disclosed men who have sex with men among self-reported heterosexual men with HIV in Southwest China: A genetic network study

**DOI:** 10.1371/journal.pone.0283031

**Published:** 2023-03-31

**Authors:** Yi Chen, Guanghua Lan, Yi Feng, Yuhua Ruan, Zhiyong Shen, Edward B. McNeil, Kailing Tang, Jinghua Huang, Yiming Shao, Mei Lin, Virasakdi Chongsuvivatwong

**Affiliations:** 1 The People’s Hospital of Guangxi Zhuang Autonomous Region and Guangxi Academy of Medical Sciences, Nanning, Guangxi, China; 2 Guangxi Key Laboratory of Major Infectious Disease Prevention and Control and Biosafety Emergency Response, Guangxi Center for Disease Control and Prevention, Nanning, Guangxi, China; 3 Faculty of Medicine, Epidemiology Unit, Prince of Songkla University, Hat Yai, Thailand; 4 State Key Laboratory of Infectious Disease Prevention and Control (SKLID), Chinese Center for Disease Control and Prevention (China CDC), Collaborative Innovation Center for Diagnosis and Treatment of Infectious Diseases, Beijing, China; Shanghai Public Health Clinical Center, Fudan University, CHINA

## Abstract

**Background:**

In Guangxi province of China, there is a high prevalence of HIV in the general population and in men who have sex with men (MSM). However, there is still a low proportion of MSM among people living with HIV. This apparent contradiction could be due to the high proportion of potential non-disclosed MSM (pnMSM) among reported heterosexual men. We analyzed the genetic linkage of HIV specimens to address this problem aiming to (1) identify the optimal genetic distance threshold, which gave the highest number of genetic clusters, (2) document the proportion of potential non-disclosed MSM (pnMSM) among self-reported heterosexual men, and (3) determine predictors for pnMSM.

**Methods:**

Pairwise genetic distances were computed among all samples. The genetic distance threshold giving the highest number of genetic clusters was identified. Self-reported heterosexual men were identified as belonging to the pnMSM group if they could be linked to any MSM in their cluster. Multinomial logistic regression was used to determine associated factors of being pnMSM.

**Results:**

The optimal genetic distance threshold was 0.75% substitutions/site. Among 896 self-reported heterosexual men, the frequency (percentage and 95% confidence interval) was 62 (6.9%, 5.2–8.6%) for pnMSM, 779 (86.9%, 84.7–89.1%) for indeterminate men and 55 (6.1%, 4.5–7.7%) for potential heterosexual men, respectively. Self-reported heterosexual men who were younger, single and more educated were more likely to be pnMSM.

**Conclusion:**

Based on these findings, there is a need to pay more attention to sexually active, young and educated self-reported heterosexual men and provide them with voluntary counselling and testing and specific HIV prevention services.

## Introduction

Guangxi is a province in south-western China and has the third highest number of HIV/AIDS cases reported at the provincial level [1]. The prevalence of HIV among men who have sex with men (MSM) reported from the Integrated Biologic and Behavioral Surveys (IBBS) increased from 0.83% in 2008 to 11.2% in 2015 [2] and then stabilized at around 10% in 2017. However, MSM contributed only 3.13% of all reported HIV/AIDS cases during 2010–2017 [3] and rose to 7.0% in 2020. These percentages were relatively low compared with neighboring provinces, which ranged from 14.4% in Chongqing to 43.5% in Hainan.

Although same-sex behaviour is not illegal in China, being homosexual or associating with MSM has negative social and cultural consequences, including rejection by family members and loss of employment [4]. Hence, stigma towards homosexual identity is common in China [5,6]. Many HIV infected MSM in China might tend to self-report as heterosexual men to avoid stigmatization [7], which could lead to an underestimation of the MSM population size. MSM who conceal their sexual identity would also miss the opportunity to access beneficial services such as MSM-specific education packages, and pre- and post-exposure prophylaxis [8].

With HIV genetic sequencing technology, pairwise genetic distances of two or more persons can be determined. With an optimal genetic distance threshold, HIV genetic clusters (or networks) can be constructed and two network members with a genetic distance less than this threshold will form a linkage. Previous studies identified potential non-disclosed men who have sex with men (pnMSM) based on whether the clusters contained MSM and self-reported heterosexual men only [9,10]. An alternative method to determine pnMSM is to use the genetic linkage, which may give a higher precision of possible transmission.

A genetic linkage between two HIV-infected people strongly implies HIV transmission between them. Furthermore, if the two people are not intravenous drug users, the linkage may imply sexual contact. Thus, if a man who reports himself as heterosexual is genetically linked to a man who has sex with other men, then he can be considered to belong to the MSM group. The aims of this study were (1) to use nucleotide sequences Pol-region and Reverse transcriptase (PR-RT) information of HIV samples obtained from self-reported heterosexual men, MSM, and women to identify the optimal genetic distance threshold, which results in the highest number of clusters (networks), (2) to document the proportion of pnMSM among self-reported heterosexual men, and (3) to determine predictors for pnMSM.

Our study provides a method to estimate the proportion of pnMSM among self-reported heterosexual men in any community where such a proportion needs to be documented.

## Materials and methods

### Ethics statement

Informed consent was waived since we used leftover specimens confirmed as HIV positive by the Western Blot test for phylogenetic analysis. The confidentiality of all study participants was maintained and their well-being was not affected. Participants’ epidemiological data were obtained by matching cases with the Guangxi HIV/AIDS case report dataset through the unique ID of the specimens. The study was approved by the ethics committee of Prince of Songkla University (REC. 63-348-18-1) and the institutional review board of the Guangxi Centre for Disease Prevention and Control (GXIRB2020-0069).

### Study setting

The study was conducted in Nanning, the capital city of Guangxi. The number of reported HIV/AIDS cases during 2016–2020 was 8463, of whom 7696 (90.9%) were reported as being infected through sexual transmission. The proportion of injecting drug use HIV/AIDS cases accounted for less than 2.0% of the total reported cases and were therefore excluded from the analysis. Among the sexual transmission cases, 6312 (82.0%) were self-reported heterosexuals and 1384 (18.0%) identified as MSM.

### Study design and study subjects

This was a cross-sectional HIV genetic-network study. In order to achieve the required sample size, we combined HIV/AIDS treatment-naïve cases diagnosed during 2016–2020 aged 18 years or older from two major types of service sites. One was from local hospitals with routinely detected HIV/AIDS cases. Most of these persons were self-reported heterosexuals. The other was from a voluntary counselling and testing clinic under the jurisdiction of the Guangxi Centre for Disease Control and Prevention where the majority of clients were MSM. These two sites combined were expected to cover over 90.0% of the HIV/AIDS reported cases in Nanning. We used leftover specimens of HIV Western Blot confirmatory test to do the genetic analysis. After removing 127 duplicates based on linkage of HIV sequence ID and citizen ID, which was conducted by other members outside of our study group, 1047 samples from the hospitals and 928 samples from the clinic were included in the phylogenetic analysis.

For HIV sequencing, the pol fragments (HXB2 positions 2253–3870, minimum length 900 bp) were amplified and sequenced using an in-house polymerase chain reaction protocol according to previously published methods [11,12]. Variables included in the analysis were age at HIV diagnosis, gender, self-reported HIV risk category, education level, marital status, occupation, ethnicity, CD4 count at diagnosis, and year of HIV diagnosis.

### Phylogenetic analysis

The nucleotide sequences PR-RT region were aligned separately using the HIV Align tool (https://www.hiv.lanl.gov/content/sequence/NEWALIGN/align.html) on the HIV Los Alamos National Laboratory database and manually adjusted using BioEdit software version 7.0.9.1 (http://www.mbio.ncsu.edu/Bioedit/bioedit.html). HIV subtypes were determined based on approximate maximum-likelihood tree construction, which was generated in IQ-TREE 2.0.6 using an ultrafast bootstrap method with 1000 iterations [13]. The best fitting model was TVM+F+R9.

The Tamura-Nei 93 (TN93) nucleotide substitution model was applied to calculate the pairwise genetic distances for all 1975 sequences across different thresholds (0.1% to 2.0%) in order to determine the optimal threshold, at which, the molecular network resolution is highest with the maximum number of clusters shown. This principle is outlined in a previous study [14] and has been applied in several recent studies [15,16]. The transmission networks were reconstructed using the HIV Transmission Cluster Engine (HIV-TRACE, http://demo.hivtrace.org/network.html), which can identify groups of putative transmission partners and assemble them in clusters regardless of HIV subtypes, potentially identifying more accurate transmission chains [17,18]. We resolved all two-way ambiguities to match their possible single character states, averaged all other ambiguities, ignored positions where either sequence has a gap, and for ambiguity rich sequences (>5% of bases are ambiguous), we averaged all resolutions [19].

The transmission clusters were used to identify pnMSM, which is the focus of this paper. We omitted the display and discussion of the phylogenetic tree to make this paper more concise.

### Identification of pnMSM

#### Cluster-based classification method

The cluster-based classification method has been described elsewhere [9,10]. Briefly, the method first chooses an optimal genetic distance threshold to group the sample into clusters whereby a member must be genetically close to at least one other member with a distance shorter than the threshold. A self-reported heterosexual man is classified as an MSM (pnMSM) if all other members in its cluster consist of MSM and self-reported heterosexual men only. A singleton is one whose HIV virus cannot be linked to any other person’s based on the genetic distance threshold.

#### Linkage-based classification method

The linkage-based method classifies a self-reported heterosexual man based on the genetic distance between his HIV and others’ in the study sample. If their genetic distance is not larger than the pre-set threshold, it would be probable that there was a transmission between them. Persons who had such close genetic distance of HIV could form a cluster or a network.

Table 1 shows the criteria of self-reported heterosexual men classification according to seven types of linkages between them and other members of the network. If a self-reported heterosexual man had ANY direct link with MSM, he was classified as pnMSM as we assumed that most of the transmission was by sexual route. If the HIV had only genetic link with women, the person was classified as “Potential heterosexual men”. Otherwise, he was classified as “Indeterminate”.

**Table 1:** Classification of self-reported heterosexual men based on who they were linked to in the same network.

Who they linked to	Classification
1. MSM only	pnMSM
2. MSM and self-reported heterosexual men	pnMSM
3. Self-reported heterosexual men only	Indeterminate
4. Women and MSM	pnMSM
5. Women only	Potential heterosexual men
6. Women and self-reported heterosexual men	Indeterminate
7. MSM, women, and self-reported heterosexual men	pnMSM
8. Singleton (not linked to any other person)	Indeterminate

MSM: Men who have sex with men.

pnMSM: Potential non-disclosed MSM.

The combination of self-reported heterosexual man with more than one type of subgroups could be seen in the network no matter linkage-based or cluster-based method were performed. However, linkage-based method could identify pnMSM from combination groups of “self-reported heterosexual men, Women and MSM” based on the linkage, while the cluster-based method could not due to it did not consider any clusters containing woman.

To make the results comparable with the cluster-based method, the threshold for the genetic distance is set to the same value. Thus, the two methods will share the same set of singletons.

#### Classification process

According to the classification criteria, we classified self-reported heterosexual men repeatedly over many rounds.

In the first round, we defined self-reported heterosexual men as pnMSM in the original network based on their linkage with any MSM. In the second and further rounds, we reclassified a self-reported heterosexual man into the pnMSM group if he could be linked with pnMSM identified from a previous round. This process continued until there were no further changes.

We compared the results from the linkage-based method with the cluster-based method to examine the extent of over- and under- estimation.

### Genetic network inference based on optimal genetic distance threshold

Both the cluster-based and linkage-based methods attempt to identify persons in clusters. The precision of the cluster definition depends on the genetic distance threshold. The shorter the distance, the stronger the evidence of transmission. However, setting a strict (too short distance) threshold will result in a higher number of singletons and more precise clusters. However, having an excessive number of singletons will not be useful for identification of pnMSM. On the other hand, relaxing the threshold will result in fewer singletons but less precise clusters. The choice of threshold, therefore, strongly affects the cluster-based method. As the cluster size is enlarged, the chance of linking a heterosexual man into an MSM cluster will increase.

The optimal threshold can give the maximum number of clusters. This will avoid too many singletons being identified and the cluster size being too large. We performed the network construction iteratively under different genetic distance thresholds (ranging from 0.001 to 0.02 substitutions/site) and plotted the changing threshold against the number of clusters and links produced to determine the optimal threshold value.

### Statistical analysis

Demographic variables were presented descriptively using frequency and percentage. The distribution of cluster types across the nodes were analyzed using the R language and environment [20]. 95% confidence intervals for estimating proportions were calculated according to *p*±1.96$\sqrt{\frac{pq}{n}}$, where *p* is the proportion of pnMSM among self-reported heterosexual men with HIV, *q* is 1–*p*, and *n* is the total number of self-reported heterosexual men with HIV in our study.

Associated factors of being pnMSM were determined using a multinomial logistic regression model with three outcome groups, namely pnMSM, potential heterosexual men and indeterminate men. We chose pnMSM as the referent group against which each of the two other groups were compared. If the degree and direction of the association between pnMSM and both groups were consistent, the predictors identified would be reasonably robust. However, in computation, when using pnMSM as the referent group, a relative risk ratio >1 would indicate that the independent variable was predicting non-pnMSM. In interpretation of the results of the regression, reverse from conventional interpretation of an odds ratio (>1 is a risk factor, <1 is a protective factor), the predictors with a relative risk ratio < 1 is actually a positive predictor.

## Results

### Basic characteristics of the study samples under optimal genetic distance threshold

S1 Table illustrates the extent of mixture for subjects recruited from the two kinds of study sites and indicates that subjects were well distributed throughout the phylogenetic tree.

S2 Table shows the risk-gender distribution of all subjects from the two kinds of study sites mixed together. The proportion of MSM overall was 33.3%.

Out of 7,696 HIV/AIDS cases diagnosed during study period and self-reported as being infected with HIV via sexual contact, 1,975 (25.7%) had left-over specimens with an adequate quantity and acceptable quality for RNA sequencing.

Table 2 compares characteristics of the study subjects. Compared to self-reported heterosexual men, MSM were younger, more educated, and had a higher proportion of singles and members of the Han ethnic group. HIV-1 subtypes were predominated by CRF07_BC among MSM whereas CRF01_AE was more common among self-reported heterosexual men. A low CD4+ count was more common among self-reported heterosexual men compared to MSM.

**Table 2:** Comparison of characteristics among women, self-reported heterosexual men and MSM.

Variables	Total	Study group		
		Women	srHM	MSM
	N = 1,975	n = 422	n = 896	n = 657
**Age group (years)**				
<30	510 (25.8)	31 (7.3)	68 (7.6)	411 (62.6)
30–39	328 (16.6)	59 (14.0)	125 (14)	144 (21.9)
40–49	287 (14.5)	68 (16.1)	145 (16.2)	74 (11.3)
50–59	306 (15.5)	107 (25.4)	180 (20.1)	19 (2.9)
≥60	544 (27.5)	157 (37.2)	378 (42.2)	9 (1.4)
**Ethnicity**				
Han	950 (48.1)	169 (40.0)	401 (44.8)	380 (57.8)
Zhuang	944 (47.8)	231 (54.7)	474 (52.9)	239 (36.4)
Other	81 (4.1)	22 (5.2)	21 (2.3)	38 (5.8)
**Education level**				
Elementary or less	622 (31.8)	243 (58.6)	367 (41.2)	12 (1.9)
Junior or high school	861 (44.1)	153 (36.9)	442 (49.7)	266 (41.0)
College or above	470 (24.1)	19 (4.6)	81 (9.1)	370 (57.1)
**Marital status**				
Single	1,101 (55.9)	137 (32.5)	412 (46.2)	552 (84.1)
Married	869 (44.1)	285 (67.5)	480 (53.8)	104 (15.9)
**Occupation**				
Employer	134 (7.4)	14 (3.4)	36 (4.1)	84 (16.2)
Employee	320 (17.7)	32 (7.7)	83 (9.5)	205 (39.4)
Farmer	894 (49.4)	268 (64.9)	597 (68.2)	29 (5.6)
Unemployed	419 (23.2)	92 (22.3)	145 (16.6)	182 (35.0)
Other	42 (2.3)	7 (1.7)	15 (1.7)	20 (3.8)
**Year of HIV diagnosis**				
2016	259 (13.1)	44 (10.4)	82 (9.2)	133 (20.2)
2017	518 (26.2)	98 (23.2)	240 (26.8)	180 (27.4)
2018	416 (21.1)	90 (21.3)	148 (16.5)	178 (27.1)
2019	389 (19.7)	97 (23.0)	207 (23.1)	85 (12.9)
2020	393 (19.9)	93 (22.0)	219 (24.4)	81 (12.3)
**HIV-1 subtype**				
CRF01_AE	888 (45.0)	238 (56.4)	433 (48.3)	217 (33.0)
CRF07_BC	572 (29.0)	63 (14.9)	198 (22.1)	311 (47.3)
CRF08_BC	330 (16.7)	109 (25.8)	213 (23.8)	8 (1.2)
CRF55_01B	113 (5.7)	4 (0.9)	31 (3.5)	78 (11.9)
Other	72 (3.6)	8 (1.9)	21 (2.3)	43 (6.5)
**CD4 count at diagnosis (cells/mm** ^**3**^ **)**				
<200	652 (36.6)	162 (38.9)	427 (48.7)	63 (12.9)
200–499	860 (48.3)	196 (47.1)	357 (40.8)	307 (62.8)
≥500	269 (15.1)	58 (13.9)	92 (10.5)	119 (24.3)
**Source of samples**				
GXCDC VCT clinic	928 (47.0)	131(31.0)	257(28.7)	540(82.2)
Hospitals	1047 (53.0)	291(69.0)	639(71.3)	117(17.8)

srMSM: Self-reported heterosexual men.

GXCDC VCT: Voluntary counselling and testing clinic under Guangxi center for disease control and prevention.

### Optimal genetic distance threshold

S1 Fig (Trend of genetic links and clusters under different TN93) illustrates the relationship between number of clusters (orange line) and number of links (blue line) on the vertical axis and TN93 genetic distance (substitutions/site) on the horizontal axis. Distances to the left indicate stricter thresholds, demanding very close similarity and fewer linkages. As the genetic distances are relaxed to allow more dissimilarity, the number of linkages increases up to the maximum on the extreme right.

A distance of 0.0075 substitutions/site was identified as the optimal genetic distance value. Using this threshold, the 1975 study subjects were grouped into 235 genetic clusters with 1085 singletons. Cluster sizes ranged from 2 to 55 as illustrated in S2 Fig (235 HIV-1 genetic clusters with 1085 singletons omitted). Among 896 self-reported heterosexual men, 379 were classified into 143 clusters with 517 singletons.

### Number of pnMSM identified

**Fig 1:**
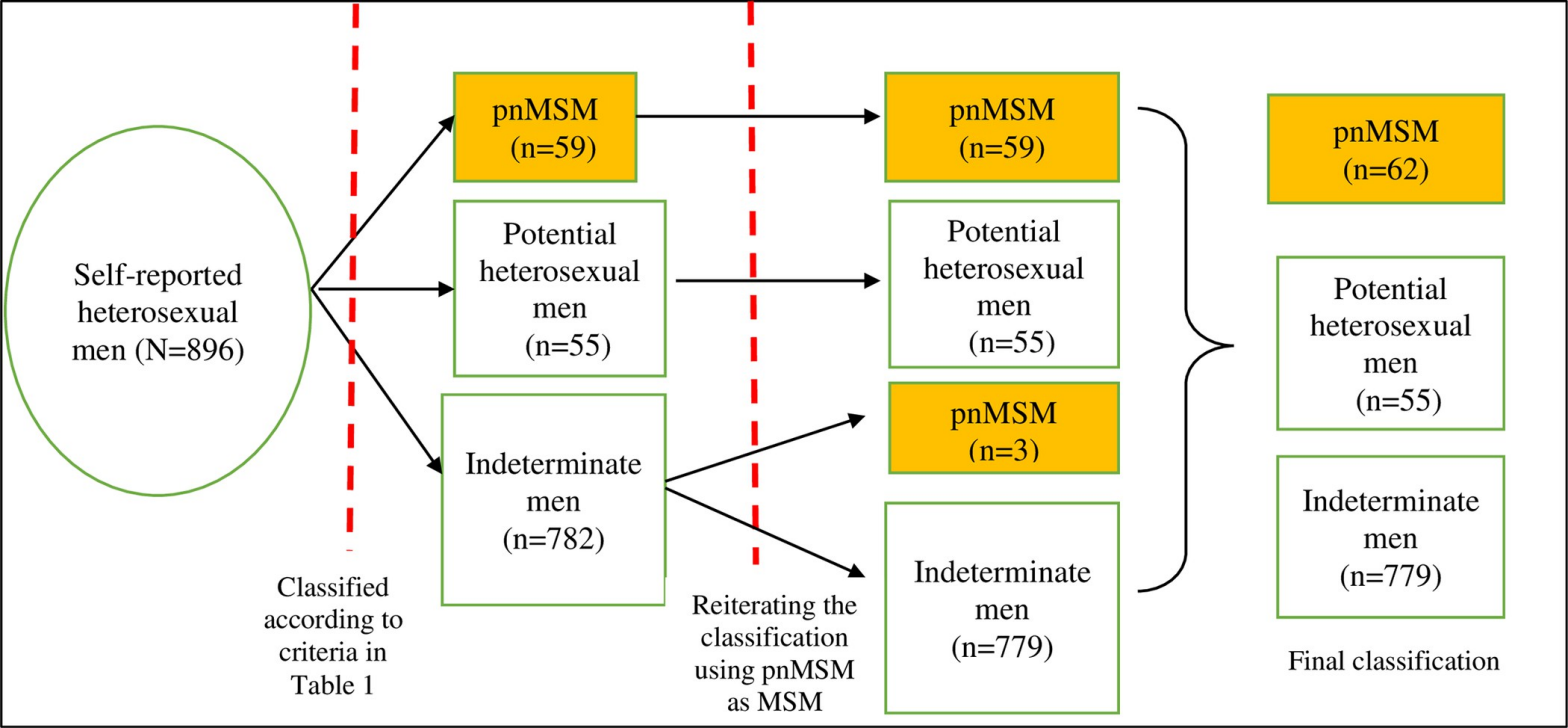
Classification of potential non-disclosed men who have sex with men (pnMSM) identified from clusters containing self-reported heterosexual men with HIV and related nodes in the genetic network using a genetic distance threshold of 0.75% substitutions/site.

Fig 1 (Classification of pnMSM identified from clusters containing self-reported heterosexual men with HIV and related nodes in the genetic network using a genetic distance threshold of 0.75% substitutions/site) summarizes the results of classification of the 896 self-reported heterosexual men. Based on the criteria shown in Table 1, there were 782 indeterminate men classified in round 1, of whom 517 were singletons. In the second round, the pnMSM classified in round 1 were assumed to be MSM and the criteria were reapplied. Three indeterminate men from the first round were reclassified as pnMSM due to their linkage with newly identified pnMSM. Reiteration of this process for the third round gave no change in the results and the process ended. Thus, eventually, there were 62 pnMSM, 55 potential heterosexual men and 779 indeterminate men. Their prevalence (95% confidence interval) were: 6.9% (5.2–8.6%), 6.1% (4.5–7.7%) and 86.9% (84.7–89.1%), respectively.

### Genetic network visualization

**Fig 2:**
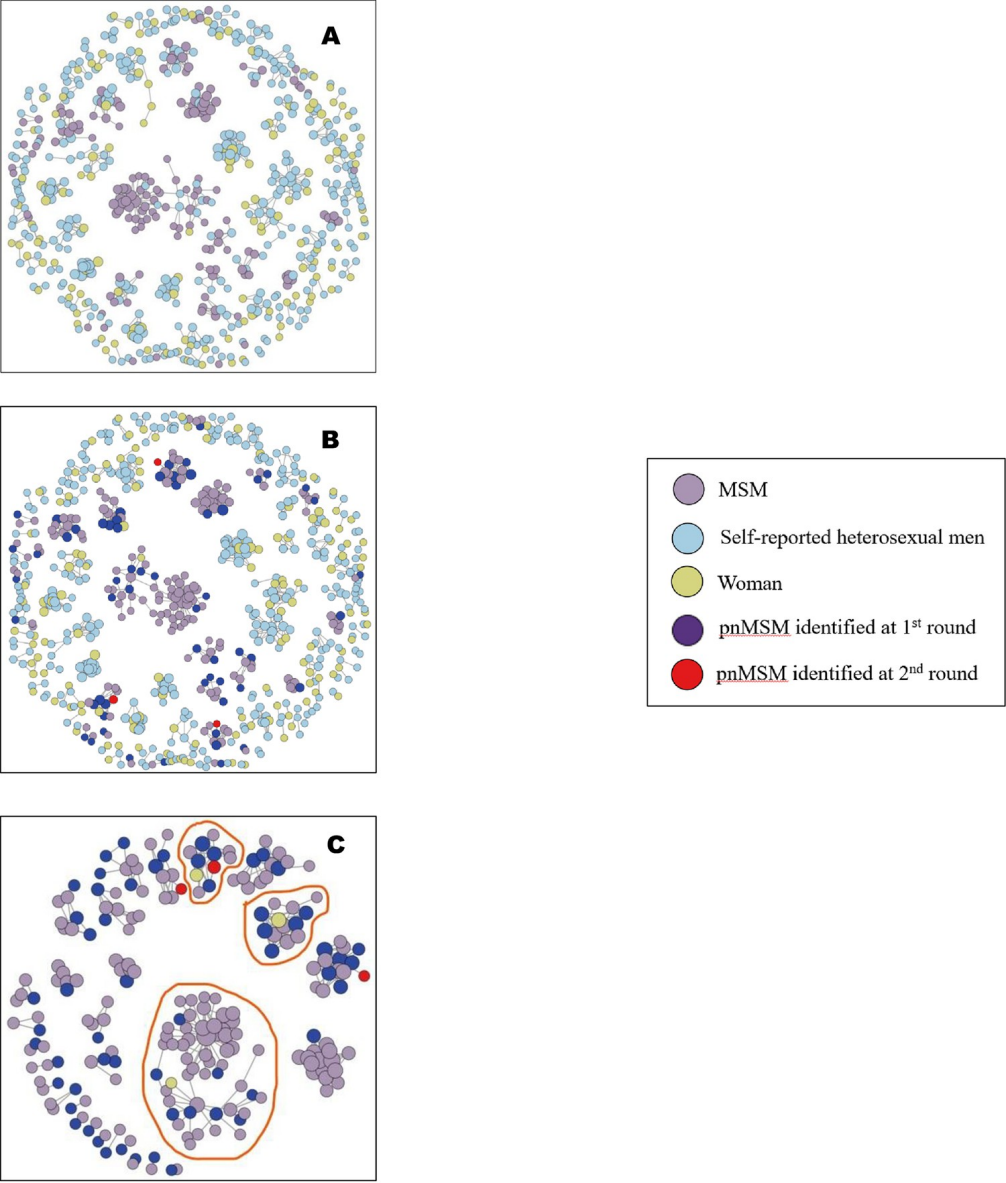
Cluster mapping of HIV-1 molecular network. 2-A is from self-reports; 2-B is from the analysis based on Fig 1 with pnMSM identified; 2-C shows 29 HIV-1 molecular clusters containing pnMSM where clusters surrounded by red loops indicate that they were comprised of self-reported heterosexual men, women, and MSM, while other clusters contained only MSM and self-reported heterosexual men. Singleton self-reported heterosexual men are omitted. **A** 143 HIV-1 molecular clusters containing self-reported heterosexual men and related nodes. **B** 143 HIV-1 molecular clusters containing self-reported heterosexual men and related nodes with potential non-disclosed men who have sex with men (pnMSM) identified (specified by dark purple and red dots). **C** 29 HIV-1 molecular clusters containing potential non-disclosed men who have sex with men (pnMSM).

To explore the connections between self-reported heterosexual men and other nodes, especially with MSM, we visualized the genetic network with clusters containing self-reported heterosexual men and related nodes. Fig 2 (Cluster mapping of HIV-1 molecular network) illustrates this genetic network including 143 molecular clusters containing 660 nodes with singletons omitted. Fig 2A (143 HIV-1 molecular clusters containing self-reported heterosexual men and related nodes) shows the nodes classified according to their self-reported risk category. Fig 2B (143 HIV-1 molecular clusters containing self-reported heterosexual men and related nodes with pnMSM identified) identified pnMSM in the first (dark purple dots, n = 59) and second (red dots, n = 3) rounds. MSM and pnMSM are confined to only 29 clusters, which are exclusively visualized in Fig 2C (29 HIV-1 molecular clusters containing pnMSM).

### Comparison of the number of pnMSM identified between linkage-based and cluster-based methods

Table 3 compares the numbers of pnMSM identified by the linkage-based and cluster-based methods. The linkage-based method gave a higher number of pnMSM for all genetic distance thresholds. S3 Table shows the details of the comparison of the number and proportion of pnMSM identified from self-reported heterosexual men from the two methods at all thresholds (ranging from 0.005 to 0.015 substitutions/site).

**Table 3:** Comparison of the number of pnMSM identified by linkage-based and cluster-based methods.

Genetic distance threshold (%)	Number of pnMSM	
	Linkage-based method	Cluster-based method
0.50	49	40
0.75	62	44
1.00	90	57
1.25	117	49
1.50	208	37

pnMSM: Potential non-disclosed men who have sex with men.

The cluster-based method identifies pnMSM in clusters of “MSM+self-reported heterosexual men”.

### Comparison between MSM and pnMSM

S4 Table compares characteristics between MSM and pnMSM identified by the linkage-based classification method. The pnMSM group were generally older, less educated, more likely to be married, and had lower CD4+ counts. In general, their background characteristics were in between self-reported heterosexual men and MSM.

### Multinomial regression analysis

Table 4 summarizes the multinomial regression model to determine associated factors for pnMSM. Since pnMSM was the baseline outcome group, relative risk ratios in this table are interpreted from the conventional case-control study. A risk ratio less than 1 indicates that the person in that specific level of the indendent variable were more likely to be pnMSM. The relative risk of being a pnMSM increased with decreasing age group. Using <40 years old as the referent group, subjects in the oldest age group were less likely to be classified as pnMSM. Similarly, other predictors for pnMSM included being single, and having a higher educational background. Other variables were not statistically significant.

**Table 4:** Multinomial logistic regression analysis comparing potential heterosexual and indeterminate men with pnMSM (n = 62).

Variables	Potential heterosexual men (n = 55)	Indeterminate men (n = 779)	p value^†^
	RRR (95% CI)	RRR (95% CI)	
Age group (years)			0.0213
<40	Ref.	Ref.	
40–49	1.05(0.29,3.74)	1.04(0.47,2.27)	
50–59	2(0.45,8.95)	2.59(0.86,7.82)	
≥60	6.11(1.45,25.64)	5.49(1.81,16.61)	
Ethnicity			0.4883
Han	Ref.	Ref.	
Zhuang	2.09(0.92,4.75)	1.41(0.77,2.56)	
Other	1(0.05,20.4)	1.35(0.15,12.2)	
Education level			0.0073
Elementary or less	Ref.	Ref.	
Junior or high school	0.32(0.1,0.99)	0.47(0.18,1.26)	
College or above	0.11(0.02,0.62)	0.17(0.05,0.52)	
Marital status			0.0002
Single	Ref.	Ref.	
Married	5.04(1.95,13.07)	1.35(0.69,2.64)	
Occupation			0.5963
Employed	Ref.	Ref.	
Unemployed	1.58(0.45,5.57)	1.17(0.56,2.44)	
Farmer	2.19(0.56,8.51)	2.29(0.86,6.08)	
Other	3.66(0.55,24.59)	1.59(0.38,6.66)	
Year of HIV diagnosis			0.9716
2016	Ref.	Ref.	
2017	0.87(0.21,3.53)	0.88(0.34,2.23)	
2018	0.96(0.21,4.38)	0.66(0.23,1.92)	
2019	0.91(0.15,5.39)	0.83(0.23,3.02)	
2020	1.4(0.27,7.22)	1.16(0.36,3.7)	
CD4 count at diagnosis (cells/m^3^)			0.3371
<200	Ref.	Ref.	
200–499	0.8(0.33,1.95)	1.27(0.65,2.49)	
≥500	0.51(0.14,1.86)	0.66(0.28,1.53)	
Sample source			0.3848
GXCDC VCT clinic	Ref.	Ref.	
Hospital routine detected HIV/AIDS cases	0.67(0.19,2.34)	1.25(0.5,3.11)	

^†^ Likelihood ratio test.

pnMSM: Potential non-disclosed men who have sex with men.

RRR: Relative risk ratio.

GXCDC VCT: Voluntary counselling and testing clinic under Guangxi centre for disease control and prevention.

## Discussion

With an optimal genetic distance threshold identified of 0.0075 substitutions/site, nearly 7% of the self-reported heterosexual men in this study were classified as pnMSM while almost 87% were indeterminate. The number of pnMSM identified by the linkage-based method was higher than those identified by the cluster-based method. The patterns of difference between pnMSM and potential heterosexual men among self-reported heterosexual men was similar with the difference between MSM and self-reported heterosexual men among the HIV positive men. MSM and the pnMSM tended to be single, young and more educated than the heterosexual men.

The HIV epidemic in Guangxi province is driven by heterosexual transmission [1,3]. The basic characteristics of self-reported heterosexual men were consistent with previous studies in Guangxi [3,21]. Self-reported heterosexual men having a low CD4 count at diagnosis indicated that they were diagnosed at a relatively late stage and demonstrates the problem of access to care among elderly groups who were infected from heterosexual contact. Previous studies found that older heterosexual men in Guangxi preferred to have high risk sexual contact with sexual workers, which caused their HIV infection [22,23]. Yet, they were rarely screened for HIV and not detected until they became seriously ill in hospital. Our study suggests that this population needs education for HIV prevention and early counselling/testing to prevent HIV infection and complications from HIV infection.

Genetic distance reflects how far away in the past two viruses had shared the same genome. A shorter distance threshold allows only recent transmissions to be detected, most of the cases will be classified as singletons, and less recent transmissions would not be detected. On the other hand, a relaxed threshold would allow past transmissions to be included in the clusters. This can dilute recent transmissions, which are more imminent to control. We chose a genetic distance threshold which gave the maximum number of clusters. The method gave a good balance within this dilemma. The 0.75% substitutions/site is equivalent to 3.5–4 years of HIV evolution [24]. Thus, the transmission among the linked cases was moderately recent.

Theoretically, the linkage-based method yields a higher probability in identifying more pnMSM subjects than the cluster-based method. This is because the presence of a woman in a cluster could simply, and maybe incorrectly, prevent self-reported heterosexual men from being classified as pnMSM even if the woman had no genetic linkage and he is actually linked with other MSM in the same cluster.

We found that pnMSM were significantly younger and well educated compared to the other groups, and had more single (unmarried) subjects than potential heterosexual men. This is consistent with the characteristics of registered MSM. Elder men were therefore less likely to have same sex practice. The difference of risk in the age groups may be the birth cohort effect where new generations of men are more prone to this type of sexual activity whereas the older generation are more accustomed to heterosexual sex with commercial sex workers.

Non-disclosure of sexual status may be due to stigma [7], which may lead to delays in receiving counselling and HIV testing. A newly developed technology for self-testing of HIV should be made available for this high-risk group while they are not ready to expose themselves to counselling and testing service. Legal measures are also needed to oppose social discrimination against HIV infection and homophobia [25]. These efforts will encourage pnMSM to disclose their same-sex behaviors and seek pre- and post-exposure prophylaxis more freely.

There are several limitations in this study. The major one was the adequacy of the leftover specimens. The missing cases could have led to incomplete analysis of the genetic linkages and led to a high number of singletons which were classified as indeterminate men. Secondly, missing cases who were men would also reduce the chance of having men in the cluster and thus biased toward the non-missing self-reported heterosexual men being classified away from pnMSM. Thirdly, the indeterminate men who were linked to both men and women would have higher likelihood of being pnMSM compared to those who were singleton. All of these limitations might lead to underestimation of pnMSM in the population. On the other hand, based on self-reporting, possible incomplete exclusion of drug users among the study men might lead to over reporting of pnMSM cases.

## Conclusions

Based on our findings, there is a need to pay more attention to sexually active, young and educated self-reported heterosexual men who are more likely to be pnMSM. Increased counselling and testing services and specific HIV preventions should be provided to this group.

## Supporting information

S1 Fig Trend of genetic links and clusters under different TN93 (Tamura and Nei 93) genetic distances. (DOCX)

S2 Fig 235 HIV-1 genetic clusters with 1085 singletons omitted. (DOCX)

S1 Table Risk-gender distribution of study samples from different sources. (DOCX)

S2 Table Extent of mixture for samples from two sources. (DOCX)

S3 Table Comparison of number and proportion of pnMSM identified by linkage-based and cluster-based methods at all genetic distance thresholds. (DOCX)

S4 Table Comparison of characteristics between MSM and pnMSM. (DOCX)

S1 File GenBank accession numbers for random selected HIV sequences. (DOCX)
