# Supplementary material for: Inferring potential non-disclosed men who have sex with men among self-reported heterosexual men with HIV in Southwest China: A genetic network study
Source: PLoS One. 2023 Mar 31;18(3):e0283031. doi: 10.1371/journal.pone.0283031 (PMC10065240; doi:10.1371/journal.pone.0283031)
Supplement: S1 Fig — (DOCX) [file pone.0283031.s001.docx]

**Supporting information**

**S1 Figure. Trend of genetic links and clusters under different TN93** (Tamura and Nei 93) genetic distances. The orange line denotes the number of clusters and the blue line denotes the number of links. The arrow designates the optimal genetic distance threshold with the maximum number of clusters.
