# Supplementary figures and images for: Inferring potential non-disclosed men who have sex with men among self-reported heterosexual men with HIV in Southwest China: A genetic network study
Source: PLoS One. 2023 Mar 31;18(3):e0283031. doi: 10.1371/journal.pone.0283031 (PMC10065240; doi:10.1371/journal.pone.0283031)

**Supporting information**


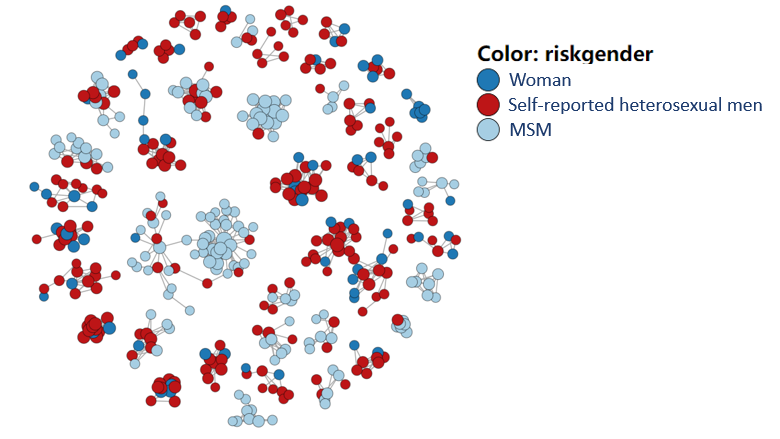


**S2 Figure.** 235 HIV-1 genetic clusters with 1085 singletons omitted

Supplement: S2 Fig — (DOCX) [file pone.0283031.s002.docx]
