# Supplementary material for: Inferring potential non-disclosed men who have sex with men among self-reported heterosexual men with HIV in Southwest China: A genetic network study
Source: PLoS One. 2023 Mar 31;18(3):e0283031. doi: 10.1371/journal.pone.0283031 (PMC10065240; doi:10.1371/journal.pone.0283031)
Supplement: S1 Table — (DOCX) [file pone.0283031.s003.docx]

**Supporting information**

**S1 Table. Extent of cluster mixture for subjects recruited from the two study sites**

| **Cluster size** | **Number of clusters** | **Number of clusters with mixture of two groups**  **n (%)** |
| --- | --- | --- |
| 2 | 137 | 13 (9.5) |
| 3-5 | 65 | 22 (33.8) |
| 6-10 | 19 | 11 (57.9) |
| 11-20 | 12 | 10 (83.3) |
| >20 | 2 | 2 (100) |
| Total | 235 | 58 (24.7) |
