# Supplementary material for: Inferring potential non-disclosed men who have sex with men among self-reported heterosexual men with HIV in Southwest China: A genetic network study
Source: PLoS One. 2023 Mar 31;18(3):e0283031. doi: 10.1371/journal.pone.0283031 (PMC10065240; doi:10.1371/journal.pone.0283031)
Supplement: S2 Table — (DOCX) [file pone.0283031.s004.docx]

**Supporting information**

**S2 Table. Risk-gender distribution of subjects recruited from the two study sites**

| **Sample source** | **srHM** | **Women** | **MSM** | **Total** |
| --- | --- | --- | --- | --- |
| GXCDC VCT clinic | 257 (27.7) | 131 (14.1) | 540 (58.2) | 928 (100) |
| Local hospitals | 639 (61.0) | 291 (27.8) | 117 (11.2) | 1047 (100) |
| Total | 896 (45.4) | 422 (21.4) | 657 (33.3) | 1975 (100) |

srHM: Self-reported heterosexual men. MSM: Men who have sex with men.

GXCDC VCT: Voluntary counselling and testing clinic under Guangxi center for disease control and prevention.

Numbers in table are frequency (%).
