# Supplementary material for: Inferring potential non-disclosed men who have sex with men among self-reported heterosexual men with HIV in Southwest China: A genetic network study
Source: PLoS One. 2023 Mar 31;18(3):e0283031. doi: 10.1371/journal.pone.0283031 (PMC10065240; doi:10.1371/journal.pone.0283031)
Supplement: S3 Table — (DOCX) [file pone.0283031.s005.docx]

**Supporting information**

**S3 Table. Comparison of number and proportion of pnMSM identified by cluster-based and linkage-based methods at all genetic distance thresholds** (No. of srHM ^2^ =896)

| **Genetic distance threshold (Substitution/Site)** | **Cluster-based**^†^ | | |  | **Linkage-based** | | | | | |
| --- | --- | --- | --- | --- | --- | --- | --- | --- | --- | --- |
|  | Number of clusters | Number of pnMSM | Proportion of pnMSM among srHM |  | 1^st^ round | 2^nd^ round | 3^rd^ round | 4^th^ round | Total No. of pnMSM | Pro. of pnMSM among srHM(%) |
| 0.50 | 29 | 40 | 4.5 |  | 47 | 2 | 0 | 0 | 49 | 5.5 |
| 0.75 | 26 | 44 | 4.9 |  | 59 | 3 | 0 | 0 | 62 | 6.9 |
| 1.00 | 32 | 57 | 6.4 |  | 83 | 7 | 0 | 0 | 90 | 10 |
| 1.25 | 27 | 49 | 5.5 |  | 109 | 8 | 0 | 0 | 117 | 13.1 |
| 1.50 | 22 | 37 | 4.1 |  | 134 | 13 | 15 | 3 | 165 | 18.4 |

pnMSM: Potential non-disclosed men who have sex with men.

srHM: Self-reported heterosexual men.

^†^Cluster-based method identifies pnMSM in clusters of MSM + srHM.
