# Supplementary material for: Inferring potential non-disclosed men who have sex with men among self-reported heterosexual men with HIV in Southwest China: A genetic network study
Source: PLoS One. 2023 Mar 31;18(3):e0283031. doi: 10.1371/journal.pone.0283031 (PMC10065240; doi:10.1371/journal.pone.0283031)
Supplement: S4 Table — (DOCX) [file pone.0283031.s006.docx]

**Supporting information**

**S4 Table. Comparison of characteristics between MSM and pnMSM**

| **Variables** | **MSM** | **pnMSM** | **P value** |
| --- | --- | --- | --- |
|  | N = 657 | N = 62 |  |
|  |  |  |  |
| Age group (years) |  |  | < 0.001 |
| <30 | 411 (62.6) | 25 (40.3) |  |
| 30-39 | 144 (21.9) | 13 (21.0) |  |
| 40-49 | 74 (11.3) | 14 (22.6) |  |
| 50-59 | 19 (2.9) | 5 (8.1) |  |
| ≥60 | 9 (1.4) | 5 (8.1) |  |
|  |  |  |  |
| Ethnicity |  |  | 0.315 |
| Han | 380 (57.8) | 35 (56.5) |  |
| Zhuang | 239 (36.4) | 26 (41.9) |  |
| Other | 38 (5.8) | 1 (1.6) |  |
|  |  |  |  |
| Education |  |  | < 0.001 |
| Elementary or less | 12 (1.9) | 6 (9.7) |  |
| Junior or high school | 266 (41.0) | 29 (46.8) |  |
| College or above | 370 (57.1) | 27 (43.5) |  |
|  |  |  |  |
| Marital status |  |  | 0.004 |
| Single | 552 (84.1) | 42 (68.9) |  |
| Married | 104 (15.9) | 19 (31.1) |  |
|  |  |  |  |
| Occupation |  |  | < 0.001 |
| Employed | 289 (55.6) | 19 (30.6) |  |
| Unemployed | 182 (35.0) | 24 (38.7) |  |
| Farmer | 29 (5.6) | 16 (25.8) |  |
| Other | 20 (3.8) | 3 (4.8) |  |
|  |  |  |  |
| Year of HIV diagnosis |  |  | 0.288 |
| 2016 | 133 (20.2) | 9 (14.5) |  |
| 2017 | 180 (27.4) | 24 (38.7) |  |
| 2018 | 178 (27.1) | 12 (19.4) |  |
| 2019 | 85 (12.9) | 8 (12.9) |  |
| 2020 | 81 (12.3) | 9 (14.5) |  |
|  |  |  |  |
| CD4 count at diagnosis (cells/m^3^) |  |  | < 0.001 |
| <200 | 63 (12.9) | 23 (37.1) |  |
| 200-499 | 307 (62.8) | 26 (41.9) |  |
| ≥500 | 119 (24.3) | 13 (21.0) |  |

pnMSM: Potential non-disclosed men who have sex with men.
