# Supplementary material for: Inferring potential non-disclosed men who have sex with men among self-reported heterosexual men with HIV in Southwest China: A genetic network study
Source: PLoS One. 2023 Mar 31;18(3):e0283031. doi: 10.1371/journal.pone.0283031 (PMC10065240; doi:10.1371/journal.pone.0283031)
Supplement: S1 File — (DOCX) [file pone.0283031.s007.docx]

**Supporting information**

**S1 file** GenBank accession numbers for random selected HIV sequences

OK011554, OK011555, OK011556, OK011557, OK011558, OK011559, OK011560, OK011561, OK011562, OK011563, OK011564, OK011565, OK011566, OK011567, OK011568, OK011569, OK011570, OK011571, OK011572, OK011573, OK011574, OK011575, OK011576, OK011577,

OK011578, OK011579, OK011580, OK011581, OK011582, OK011583, OK011584, OK011585,

OK011586, OK011587, OK011588, OK011589, OK011590, OK011591, OK011592, OK011593,

OK011594, OK011595, OK011596, OK011597, OK011598, OK011599, OK011600, OK011601,

OK011602, OK011603, OK011604, OK011605, OK011606, OK011607, OK011608, OK011609, OK011610, OK011611, OK011612, OK011613, OK011614, OK011615, OK011616, OK011617,

OK011618, OK011619, OK011620, OK011621, OK011622, OK011623, OK011624, OK011625,

OK011626, OK011627, OK011628, OK011629, OK011630, OK011631, OK011632, OK011633,

OK011634, OK011635, OK011636, OK011637, OK011638, OK011639, OK011640, OK011641,

OK011642, OK011643, OK011644, OK011645, OK011646, OK011647, OK011648, OK011649,

OK011650, OK011651, OK011652, OK011653, OK011654, OK011655, OK011656, OK011657,

OK011658, OK011659, OK011660, OK011661, OK011662, OK011663, OK011664, OK011665,

OK011666, OK011667, OK011668, OK011669, OK011670, OK011671, OK011672, OK011673,

OK011674, OK011675, OK011676, OK011677, OK011678, OK011679, OK011680, OK011681,

OK011682, OK011683, OK011684, OK011685, OK011686, OK011687, OK011688, OK011689,

OK011690, OK011691, OK011692, OK011693, OK011694, OK011695, OK011696, OK011697,

OK011698, OK011699, OK011700, OK011701, OK011702, OK011703, OK011704, OK011705,

OK011706, OK011707, OK011708, OK011709, OK011710, OK011711, OK011712, OK011713,

OK011714, OK011715, OK011716, OK011717, OK011718, OK011719, OK011720, OK011721,

OK011722, OK011723, OK011724, OK011725, OK011726, OK011727, OK011728, OK011729,

OK011730, OK011731, OK011732, OK011733, OK011734, OK011735, OK011736, OK011737,

OK011738, OK011739, OK011740, OK011741, OK011742, OK011743, OK011744, OK011745,

OK011746, OK011747, OK011748, OK011749, OK011750, OK011751, OK011752, OK011753,

OK011754, OK011755, OK011756, OK011757, OK011758, OK011759, OK011760, OK011761,

OK011762.
